# Chronic Disease Surveillance Systems Within the US Associated Pacific Island Jurisdictions

**Published:** 2011-06-15

**Authors:** Gwen Hosey, Henry Ichiho, Dawn Satterfield, Irene Dankwa-Mullan, Stevenson Kuartei, Kyu Rhee, Tayna Belyeu-Camacho, Ione deBrum, Yorah Demei, Kipier Lippwe, Patrick Solidum Luces, Faiese Roby

**Affiliations:** Centers for Disease Control and Prevention. Ms Hosey is a doctoral student at the Uniformed Services University of the Health Sciences, Bethesda, Maryland; Papa Ola Lökahi, Pacific Diabetes Education Program, Honolulu, Hawaii; Centers for Disease Control and Prevention, Atlanta, Georgia; National Institute on Minority Health and Health Disparities, Bethesda, Maryland; Ministry of Health, Koror, Republic of Palau; Health Resources and Services Administration, Rockville, Maryland; Saipan, Commonwealth of the Northern Mariana Islands; Diabetes Prevention and Control Program Coordinator, Republic of the Marshall Islands; Noncommunicable Disease Administrator, Koror, Palau; Noncommunicable Disease and Lifestyle Program Manager, Palikir, Pohnpei, Federated States of Micronesia; Diabetes Prevention and Control Program Coordinator, Hagatna, Guam; Diabetes Prevention and Control Program Coordinator, Pago Pago, American Samoa

## Abstract

In recent years, illness and death due to chronic disease in the US Associated Pacific Islands (USAPI) jurisdictions have dramatically increased. Effective chronic disease surveillance can help monitor disease trends, evaluate public policy, prioritize resource allocation, and guide program planning, evaluation, and research. Although chronic disease surveillance is being conducted in the USAPI, no recently published capacity assessments for chronic disease surveillance are available. The objective of this study was to assess the quality of existing USAPI chronic disease data sources and identify jurisdictional capacity for chronic disease surveillance. The assessment included a chronic disease data source inventory, literature review, and review of surveillance documentation available from the web or through individual jurisdictions. We used the World Health Organization's Health Metric Network Framework to assess data source quality and to identify jurisdictional capacity. Results showed that USAPI data sources are generally aligned with widely accepted chronic disease surveillance indicators and use standardized data collection methodology to measure chronic disease behavioral risks, preventive practices, illness, and death. However, all jurisdictions need to strengthen chronic disease surveillance through continued assessment and expanded support for valid and reliable data collection, analysis and reporting, dissemination, and integration among population-based and institution-based data sources. For sustained improvement, we recommend investment and technical assistance in support of a chronic disease surveillance system that integrates population-based and institution-based data sources. An integrated strategy that bridges and links USAPI data sources can support evidence-based policy and population health interventions.

## Introduction

Although chronic disease has long concerned high-income countries, 80% of chronic disease deaths occur in low- to middle-income countries (1). A complex interplay of socioeconomic, demographic, technologic, cultural, environmental, and biological factors explains this epidemiologic transition from communicable disease to noncommunicable disease (NCD) (1,2). The burden of chronic disease is substantial in the US Associated Pacific Islands (USAPI) jurisdictions (American Samoa, Guam, Commonwealth of the Northern Mariana Islands [CNMI], Federated States of Micronesia [FSM] [Chuuk, Kosrae, Pohnpei, and Yap], Republic of Palau, and Republic of the Marshall Islands [RMI]). For example, although differences in age-standardizations hinder comparison (3,4), age-standardized cardiovascular disease (CVD) mortality estimates among the USAPI jurisdictions are generally higher than similar US age-standardized CVD mortality estimates (5,6) (Table 1).

The USAPI jurisdictions' population spreads across 104 inhabited islands in more than 3 million square miles of ocean, crossing the International Date Line. Despite the challenges of geographic isolation, dependence on US and international aid, and lack of health care funding (7), the USAPI jurisdictions are targeting chronic disease prevention by developing partnerships and approaches that reflect the interface between Pacific cultures and Western science (2,8,9). In addition, a May 2010 Pacific Island Health Officers Association resolution declaring a state of health emergency due to the epidemic of chronic disease encourages stronger coordination of partnerships across multiple sectors to mobilize policy, investments, and technical resources to reduce the prevalence and costs of chronic disease within the region (10). Effective chronic disease surveillance systems can support this effort and help the USAPI leadership monitor disease trends, evaluate public policy, prioritize resource allocations, and guide program planning, evaluation, and research.

Surveillance is defined as the ongoing, systematic collection, analysis, interpretation, and dissemination of data essential for health promotion and disease prevention (11). Chronic disease surveillance data sources supported by the Centers for Disease Control and Prevention (CDC), World Health Organization (WHO), and USAPI health care systems can be divided into 2 main categories: population-based (ie, household surveys) and institution-based (ie, disease registries) (Figure). The objective of this study was to assess the quality of existing USAPI chronic disease data sources and identify the capacity for chronic disease surveillance by individual jurisdiction, following WHO's Health Metric Network Framework (HMNF). We also offer recommendations for continued capacity building to strengthen surveillance within the region.

**Figure 1:**
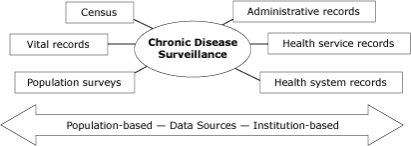
Potential chronic disease surveillance data sources. Chronic disease surveillance may include both population-based and institution-based data sources. Population-based sources include census data, vital records, and population health surveys. Institution-based sources include administrative records (eg, tax revenues), health service records (eg, occupational health), and health system records (eg, disease registries).Adapted from Health Metrics Network Framework (http://www.who.int/healthmetrics/documents/hmn_framework200803.pdf).

## Methods

After reviewing the literature and documents available on the web or through individual jurisdictions, we established an inventory of data sources for monitoring standard chronic disease indicators (11). Using this list, USAPI chronic disease representatives identified their jurisdictions' existing data sources, frequency of data collection, and availability of analysis and reporting. For each identified data source, the frequency of data collection, analysis, and reporting was confirmed by the respective USAPI Ministry of Health or Department of Health administration. CDC verified USAPI participation in the CDC-supported data sources. The US Renal Data System (USRDS) Western Pacific Renal Network verified the Medicare certification dates for jurisdictions participating in the USRDS data network (S. Tanner, oral communication, April 2011).

We completed a literature search concerning USAPI capacity for chronic disease surveillance through the National Library of Medicine’s and National Institutes of Health’s PubMed (www.ncbi.nlm.nih.gov/pubmed/) and EBSCO’s Cumulative Index to Nursing and Allied Health Literature (CINAHL). Publications included meet the following criteria: 1) they assessed chronic disease surveillance capacity for the USAPI region overall or by individual jurisdiction and 2) were published in English within the last 10 years (ie, 1999 through October 2010). We excluded publications that presented only results of USAPI chronic disease-related research (ie, epidemiologic, clinical, and qualitative) or program evaluation without inclusion of an assessment related to chronic disease surveillance in the region overall or by individual jurisdiction. Search terms were “chronic disease,” “population surveillance,” “disease surveillance,” “American Samoa,” “Guam/epidemiology,” “Micronesia/epidemiology,” and “Marshall Islands.” To select the publications, the titles and abstracts were reviewed by 1 author (G.H.) for relevance; those that met the inclusion criteria were selected. Subsequently, the entire text of selected publications was read and publications that did not meet the inclusion criteria were excluded.

We used the WHO HMNF as the foundation for assessing the quality of USAPI chronic disease surveillance data sources and identifying jurisdiction capacity (Table 2). The HMNF is a global health partnership formed in 2005 that supports assessment and capacity building for health information systems in low- to middle-income countries (12). Two authors (G.H., H.I.) completed independent assessments for each data source by using supporting documentation retrieved from web-based archives, literature review, or from surveillance documentation provided by individual jurisdictions. Aggregate scores for each assessment criterion ranged from 3 (highly adequate) to zero (not adequate). We did not score data sources without complete documentation.

## Results

USAPI data sources are listed by category and data availability by year (Table 3). A summary description of each data source follows.

### Population-based data sources

The Behavioral Risk Factor Surveillance System (BRFSS) is a standardized survey that includes measures for chronic disease risk factors and conditions, preventive health practices, and access to health care among adults (≥18 y) (www.cdc.gov/brfss/index.htm). The BRFSS uses a computer-assisted telephone-interviewing system with participation limited to households with available telephone service.

The WHO STEPwise approach (STEPS) is a standardized population health survey of adults aged 25 to 64 years that measures chronic disease and associated risk factors. STEPS involves a 3-step sequential process: 1) a questionnaire assessing demographic, behavioral, and lifestyle risks; 2) direct anthropometrical and blood pressure measures; and 3) biochemical assessment of blood samples www.who.int/chp/steps/en). 

The Youth Risk Behavior Surveillance System (YRBSS) is a standardized national school-based surveillance system that surveys students in grades 9 through 12 to measure behaviors that contribute to unintentional injuries and violence; tobacco use; alcohol and drug use; unintended pregnancy and sexually transmitted diseases; unhealthy dietary behaviors; and physical inactivity. Participating USAPI jurisdictions conduct the survey every 2 to 4 years (www.cdc.gov/yrbss).

The Youth Tobacco Survey (YTS) is a standardized school-based survey that provides data necessary to support the design, implementation, and evaluation of tobacco prevention and control programs for students in middle (grades 6-8) and high (grades 9-12) schools. The similar Global Youth Tobacco Survey (GYTS) is a standardized school-based survey (students aged 13-15 years) designed to build global capacity to monitor youth tobacco use, guide implementation and evaluation of tobacco prevention and control programs, and compare tobacco use data (www.cdc.gov/tobacco/index.htm). USAPI jurisdictions generally participate in the YTS every 4 to 5 years. Each USAPI jurisdiction may conduct the YTS or the GYTS.

### Institution-based data sources

**Disease registries**

**Cancer.** Each jurisdiction collects data on cancer incidence, types, and sites and extent of cancer at diagnosis. USAPI jurisdictions submit their cancer data to the Pacific Regional Central Cancer Registry, which compiles and reports them to CDC as de-identified cancer information (www.cdc.gov/cancer/npcr).

**Renal disease.** USRDS is a national data system that collects, analyzes, and distributes information about end-stage renal disease in the United States (www.usrds.org). Medicare-certified dialysis centers operating in the USAPI contribute data to USRDS. Guam has 4 certified facilities submitting data; American Samoa has 1 certified facility and the CNMI has 2 certified facilities. The USRDS does not include FSM, Palau, or RMI (because of federal compact agreements).

**Health system records**

Vital statistics include data on births and deaths (including fetal deaths). For chronic disease surveillance, mortality data are used to track underlying and contributing cause of death and life expectancy. Using standard forms and procedures (ie, International Classification of Diseases, 10th revision [ICD-10] codes to classify deaths), the National Vital Statistics System (NVSS) within the National Center for Health Statistics estimates mortality rates for American Samoa, CNMI, and Guam (5). Mortality estimates for FSM, Palau, and RMI are available through WHO (6) or individual jurisdictions.

Hospital discharge data are abstracted records documenting an individual's hospital stay. These records include information on patient demographics, diagnosis, treatment, and discharge condition. Individual jurisdictions collect, analyze, and report hospital discharge data.

### Literature review data sources

The MEDLINE and CINAHL database search found no published reports regarding assessment of chronic disease surveillance capacity within the USAPI region overall. Haddock (13) provided a historical perspective of Guam's communicable disease, vital statistics, cancer, and maternal-child health surveillance. Most published reports were specific to assessment of the USAPI's capacity for cancer control and prevention (including surveillance), summarized by Tsark and Braun (14) and published in *Pacific Health Dialog* (15).

### Assessment of USAPI data sources

The following summarizes data source assessments (Table 4).

**Content and scope**

In general, the quality of data source content was rated as highly adequate; most jurisdictions reported  participation in standardized population-based surveys (ie, BRFSS, YRBSS, GYTS, YTS, or STEPS). Jurisdictions also reported continual administration of vital records, disease registries (eg, cancer, USRDS), and other health system records (eg, hospital discharge). Collectively, these data sources provide most jurisdictions with uniform measures for cancer, CVD, diabetes, tobacco and alcohol use, physical activity and nutrition, other diseases and risk factors, demographics, and socioeconomic conditions.

Data collection frequency and the availability of a nationally representative population-based sample vary among jurisdictions. For example, the annual Guam BRFSS uses a representative adult (≥18 y) population sample. In comparison, STEPS uses a sample of representative adults (aged 25-64 y), although individual jurisdictions may adjust the age range of the target population. However, administration of the STEPS survey ranges from every 5 years in Pohnpei (2002 and 2007) to more than 5 years for American Samoa (2004) and RMI (2002). Finally, whereas representative data were consistently available for Palau's YRBS, availability of representative YRBS data from other jurisdictions varied by survey year.

**Jurisdictional capacity**

Generally, jurisdictional capacity is adequate; jurisdictions rely on US or WHO fiscal, administrative, and technical support for most data sources. Except for the STEPS survey, most USAPI population-based surveys have weighted data available by subgroup (ie, age, sex) across survey years. However, disaggregated analysis by socioeconomic status (ie, income, education, and occupation) is limited to selected measures within the Guam BRFSS. Availability of STEPS disaggregated analysis by socioeconomic status is unavailable for participating jurisdictions.

**Dissemination**

Dissemination of metadata and microdata files varied across data sources. For example, dissemination of the BRFSS, YRBSS, GYTS, and YTS data analysis and reports was adequate, supported by the availability of reports within 12 months of survey completion and metadata accessible through CDC websites. Microdata are available on request or through web-based data repositories. In comparison, dissemination of STEPS data analysis and reports range from present but not adequate (FSM and RMI) (16,17) to adequate (American Samoa) (18). Although STEPS metadata and microdata are available through the STEPS website or on request, the timeliness of analysis and reporting varies across participating jurisdictions.

For USAPI jurisdictions participating in the USRDS, annual reports and a web-based data repository are publicly available. USAPI mortality estimates, supported by the NVSS and WHO, are timely (ie, <4 y), with metadata and microdata available (5,6). USAPI jurisdictions generate hospital discharge reports that are available to jurisdiction health program administrators and planners, physicians, and others on request.

Additionally, the WHO Western Pacific Office and Secretariat of the Pacific Community (SPC) provide web-based links to current country health profiles (19) and the Pacific Regional Information System databanks (20) for each USAPI jurisdiction. The WHO databanks, updated annually, contain mostly crude data supplied by jurisdictions or compiled from national surveys, reports, policy documents, and databases. The PRISM databank links to jurisdiction statistical websites and provides regional data tables. However, data availability, coverage, and reliability vary from jurisdiction to jurisdiction and for each reported measure. For example, FSM and RMI are the sole jurisdictions that link (PRISM) to detailed reports related to mortality and hospital discharge summaries (20).

**Integration**

Overall, integration of available surveillance reports by USAPI chronic disease teams was rated as adequate across jurisdictions. For example, jurisdictions have used surveillance reports to develop a multiyear plan (2009-2013) supported by CDC for the integration of tobacco control and diabetes prevention and control programs, although the incorporation of available population-based data is generally more extensive than for institution-based chronic disease data sources. The USAPI are linking the multiyear plans for integrated tobacco control and diabetes prevention and control with other NCD prevention initiatives to establish holistic approaches, decrease program overlap, and leverage resources within the islands.

CNMI, FSM, Palau, and RMI, with support from WHO and SPC, have developed national NCD plans (2008-2011), focused on reducing behavioral risk factors (eg, tobacco and alcohol use, dietary behaviors, and physical inactivity). American Samoa and Guam began development of national NCD plans in 2010, linking a number of healthy lifestyle initiatives (eg, Live Healthy Guam) (21) and comprehensive cancer control plans: Guam Comprehensive Cancer Control Plan (2007-2012) and American Samoa Cancer Prevention Plan (2001-2012) (9). Because most of the USAPI NCD multiyear plans were developed within the last 2 to 3 years, use of surveillance data in program evaluation was not assessed.

## Discussion

Our review showed that the USAPI jurisdictions are using both population-based and institution-based data sources to build capacity for chronic disease surveillance. The USAPI chronic disease data sources are aligned with widely accepted indicators for chronic disease surveillance (11) that use standardized measures and methodology to collect, analyze, and report data related to chronic disease behavioral risk, preventive practices, illness, and death. Consistent use of these data sources allows the USAPI to establish population benchmarks, compare chronic disease trends regionally and among other population groups within the United States and internationally, set priorities for resource allocations, and guide evidence-based policy and population health interventions needed for chronic disease prevention.

However, the review also illustrated the need to strengthen USAPI chronic disease surveillance through expanded support for valid and reliable data collection, analysis, and reporting among population-based data sources. For example, 5 jurisdictions began monitoring and tracking health risk behaviors among youth and young adults (YRBSS, YTS) in the early 1990s. Although these surveys are administered at standard intervals, statistical analysis and reports are generated by CDC for participating jurisdictions that obtain an overall response rate of 60% or more and submit appropriate survey documentation. With the exception of Palau's YRBSS, timely and reliable data collection representative of the target YRBSS youth and young adult population across survey years varies among participating jurisdictions. Similar challenges exist with obtaining representative samples every 4 to 5 years for the YTS and GYTS.

Additionally, Guam, through participation in BRFSS, is the sole jurisdiction with sustained capacity for monitoring and tracking adult (≥18 y) health risk behavior and preventive practices. Other jurisdictions (eg, Palau) are building capacity for participation in BRFSS or STEPS (American Samoa, CNMI, FSM, and RMI), but financial resources and organizational capacity are limited. These constraints were particularly evident for STEPS, resulting in challenges with timeliness and consistency of survey administration (>5 y in American Samoa and RMI), data analysis (ie, standardized weighting and disaggregation by age, sex, and socioeconomic status), and reporting. USAPI jurisdictions' chronic disease surveillance infrastructure does not adequately support the standard and complex analysis of STEPS and other available surveillance data.

Although jurisdictional institution-based chronic disease data sources use nationally accepted standards and methodology, data quality concerns remain. These concerns are

- Timeliness of data collection, analysis, and reporting.
- Underreported vital statistics registration data.
- Underreported diagnostic or mortality data for USAPI residents who receive medical treatment in the US mainland.
- Systematic biases in diagnosis by health care providers in islands or atolls with limited medical support.
- Health record system issues that include challenges with broadband Internet access, lack of electronic medical record systems, lack of synthesis or analysis across multiple record systems, incorrect or incomplete death certificates, misinterpretation of ICD rules, and variations of coding categories for unknown and ill-defined diseases or cause of death (5,6,14).

Finally, this review did not assess biases within population-based data sources. For instance, the YRBSS and YTS exclude youths not attending or registered within jurisdictional school systems. The BRFSS includes only households with an available landline telephone and uses self-reported data. Additionally, sample size in some survey modules may limit data analysis. Lastly, responders to population health surveys might not be representative of the total target population.

## Conclusion

Chronic disease surveillance can provide a foundation for population health efforts designed to address health disparities within USAPI communities. Using the HMNF, this assessment provides an initial platform to understand the quality of existing USAPI data sources and identify jurisdictional capacity for chronic disease surveillance. The need to strengthen USAPI chronic disease surveillance through continued assessment and expanded support for valid and reliable data collection, analysis and reporting, dissemination, and integration among population-based and institution-based data sources is common across jurisdictions. Continued engagement of USAPI leadership across multiple sectors (eg, public health, business, education, faith- and community-based groups) to empower innovative systems and linkages for chronic disease surveillance is essential for understanding and improving health within Pacific communities.

Our recommendations are aligned with the White House Initiative on Asian Americans and Pacific Islanders (www.whitehouse.gov/administration/eop/aapi) and the Department of Health and Human Services (HHS) National Partnership for Action to End Health Disparities (www.minorityhealth.hhs.gov/npa/) designed to mobilize a comprehensive, community-driven, and sustained approach to reducing health disparities among racial and ethnic minorities. Recommendations include continued assessment, investment, and technical assistance in support of a chronic disease surveillance system that integrates USAPI population-based and institution-based data sources. Innovative strategies that link and expand these data sources could advance evidence-based policy and environmental transformations that target chronic disease prevention. Related recommendations include 1) collaboration among USAPI governance, local and regional partnerships, and US and international agencies to integrate surveillance; 2) investments to strengthen USAPI infrastructure that support an expanded surveillance system; 3) workforce development, through education and training, to promote quality surveillance; and 4) translation of data to inform policy, research, and program planning and evaluation at local, national, and international levels.

## Figures and Tables

**Table 1:** Age-Adjusted Estimates of Cardiovascular Disease Death Rates per 100,000 Population in the United States and US Associated Pacific Islands

Location	NCHS CVD^a^ Death Rate Estimates 2007^b^	**WHO CVD^a^ Death Rate Estimates 2004** c
United States	233	179
American Samoa	372	NA
Guam	278	NA
CNMI^d^	167	NA
RMI	NA	502
Palau	NA	390
FSM	NA	364

Sources: Xu et al (5) and World Health Organization Statistical Information Systems (6)

Abbreviations: CVD, cardiovascular disease; NA, no estimate available; CNMI, Commonwealth of the Northern Mariana Islands; RMI, Republic of the Marshall Islands; Palau, Republic of Palau; FSM, Federated States of Micronesia.

a CVD includes rheumatic, hypertensive, ischemic, cerebrovascular, inflammatory, and other forms of heart disease.

b Age-standardized to the US population census per 100,000 population, 2007.

c Age-standardized by using WHO World Standard methodology per 100,000 population, 2004.

d CVD estimate excludes cerebrovascular disease.

**Table 2:** Assessing Chronic Disease Surveillance Data Source Quality — Criterion, Definition, and Rating Scale

Core Assessment Criterion	Definition	Rating Scale			

		Highly adequate	Adequate	Present But Not Adequate	Not Adequate

		3	2	1	0
**Content and scope**	Content and scope includes 1) standard chronic disease indicators^a^ 2) representative population or reliable health record system or both 3) administration frequency ≤5 years	Meets all criteria	Meets 2 criteria	Meets 1 criterion	Frequency >6 years
**Jurisdiction capacity**	Capacity for 1) survey administration: sample design and field work; data processing; and analysis and/or health recording by using ICD-10 coding; data processing and analysis 2) disaggregated analysis by age, sex, locale 3) disaggregated analysis by socioeconomic position: education and income (as appropriate) 4) follows standards for consent, confidentiality, and data access protection	Capacity for all criteria	Capacity for 3 criteria	Capacity for 1-2 criteria	No evidence
**Dissemination**	Availability of 1) summary reports within 1-4 years after completion of survey or health record data collection 2) metadata^b^ publicly available 3) microdata^c^ available	Availability of all criteria	Availability of 2 criteria	Availability of 1 criterion	No evidence
**Integration**	Jurisdiction chronic disease team: 1) uses available data reports to support an integrated multiyear chronic disease prevention plan 2) works across chronic disease programs to coordinate and strengthen surveillance efforts 3) uses surveillance data for program planning and evaluation	Meets all criteria	Meets 2 criteria	Meets 1 criterion	No evidence

Source: World Health Organization (12).

Abbreviation: ICD-10, International Classification of Diseases, 10th revision.

a Chronic disease indicators are divided into 8 categories, representing chronic disease conditions, risk factors, and social context: cancer, cardiovascular disease, diabetes, arthritis, tobacco and alcohol use, physical activity and nutrition, other diseases and risk factors, and overarching conditions (eg, socioeconomic, life expectancy, and health insurance). Centers for Disease Control and Prevention, 2004 (11).

b Metadata is defined as structured information that describes, locates, and helps retrieve data resource (includes design, sampling methodology, and questionnaires).

c Microdata is defined as survey data set (results).

**Table 3:** Chronic Disease Surveillance Sources and Availability, US Associated Pacific Islands

Core Assessment Criterion	American Samoa	CNMI	FSM				Guam	Republic of Palau	RMI

			Chuuk	Kosrae	Pohnpei	Yap			
**Population-based^a^**									
**BRFSS**									
Availability by year	NA	2009^b^	NA	NA	NA	NA	2001-2003; 2007-2011	2009^c^	NA
**WHO STEPS**									
Availability by year	2004	2010^d^	2005^e^	2009^e^	2002, 2007^e^	2009^e^	NA	NA	2002
**YRBSS**									
Availability by year	1993, 1997, 1999, 2007	2003, 2005, 2007, 2009	NA	NA	NA	NA	1995, 1997, 2001, 2007	1999, 2001, 2003, 2005, 2007, 2009	2003, 2007, 2009
**YTS**									
Availability by year	2005	2000, 2004	2000	2000	2000	NA	2002	2000, 2005, 2009	NA
**GYTS**									
Availability by year	2010^d^	2010^d^	2007^f^	2007^f^	2007^f^	2007^f^	NA	NA	2009
**Institution-based**									
**Disease registries**									
**Cancer Registry**									
Availability by year	2007-ongoing	2007-ongoing	2007^f^-ongoing	2007^f^-ongoing	2007^f^-ongoing	2007^f^-ongoing	2007-ongoing	1999-ongoing	2007-ongoing
**US Renal Data System (USRDS)**									
Availability by year	1982-ongoing	1983-ongoing	NA	NA	NA	NA	1977-ongoing	NA	NA
**Health record data systems**									
**Vital Statistics**									
Availability	Ongoing	Ongoing	Ongoing^f^	Ongoing^f^	Ongoing^f^	Ongoing^f^	Ongoing	Ongoing	Ongoing
**Hospital Discharge**									
Availability	Ongoing	Ongoing	Ongoing^f^	Ongoing^f^	Ongoing^f^	Ongoing^f^	Ongoing	Ongoing	Ongoing

Abbreviations: CNMI, Commonwealth of the Northern Mariana Islands; FSM, Federated States of Micronesia; RMI, Republic of the Marshall Islands, BRFSS, Behavioral Risk Factor Surveillance System; NA, not applicable; WHO STEPS, World Health Organization STEPwise Approach; YRBSS, Youth Risk Behavior Surveillance System; YTS, Youth Tobacco Survey; GYTS, Global Youth Tobacco Survey.

a Weighted data available unless otherwise indicated.

b Independent administration of cross-sectional household interview using BRFSS questionnaire supported through CNMI Department of Public Health.

c Standardized BRFSS point-in-time survey.

d Anticipates completion of data collection in 2011.

e Unweighted data only.

f Participates under FSM National Health Statistics Office.

**Table 4:** Summary of USAPI Chronic Disease Surveillance Data Source Quality by Jurisdiction^a^

Data Source	American Samoa	CNMI	FSM				Guam	Republic of Palau	RMI

			Chuuk	Kosrae	Pohnpei	Yap			
**BRFSS**									
Content/scope	NS	NA^b^	NS				3	NA^c^	NS
Jurisdiction capacity							3		
Dissemination							3		
Integration							2		
**WHO STEPS**									
Content/scope	0	NA^d^	NA^e^	NA^e^	3^f^	NA^e^	NS	NS	0
Jurisdiction capacity	2				2^f^				2
Dissemination	3				2^f^				2
Integration	3				3^f^				3
**YRBSS**									
Content/scope	2	2	NS				2	3	2
Jurisdiction capacity	3	3					3	3	3
Dissemination	3	3					3	3	3
Integration	2	2					2	2	2
**GYTS or YTS**									
Content/scope	2	2	2				0	3	2
Jurisdiction capacity	2	2	2				2	2	2
Dissemination	2	2	2				2	2	2
Integration	2	2	2				2	2	2
**Cancer registry**									
Content/scope	3	3	3				3	3	3
Jurisdiction capacity	NA^g^	NA^g^	NA^g^				NA^g^	NA^g^	NA^g^
Dissemination									
Integration									
**US Renal Data System (USRDS)**									
Content/scope	3	3	NS				3	NS	NS
Jurisdiction capacity	2	2					2		
Dissemination	2	2					2		
Integration	1	1					1		
**Vital statistics**									
Content/scope	2	2	2				2	2	2
Jurisdiction capacity	2	2	2				2	2	2
Dissemination	2	2	2				2	2	2
Integration	1	1	1				1	1	1
**Hospital discharge**									
Content/scope	NA^g^	2	2				2	2	2
Jurisdiction capacity		2	2				2	2	2
Dissemination		1	1				1	1	1
Integration		1	1				1	1	1

Abbreviations: CNMI, Commonwealth of the Northern Mariana Islands; FSM, Federated States of Micronesia; RMI, Republic of the Marshall Islands; BRFSS, Behavioral Risk Factor Surveillance System; NS, no survey was conducted; NA, not assessed; WHO STEPS, World Health Organization STEPwise Approach; YRBSS, Youth Risk Behavior Surveillance System; GYTS, Global Youth Tobacco Survey; YTS, Youth Tobacco Survey.

a Key: 3, highly adequate; 2, adequate; 1, present but not adequate; 0, not adequate.

b Independent administration of BRFSS questionnaire in 2009; data analysis and reporting in process; assessment not completed.

c BRFSS point-in-time survey; data analysis and reporting not available; assessment not completed.

d Anticipates STEPS data collection completion in 2011; assessment not completed.

e STEPS data analysis and reporting not available; assessment not completed.

f Assessment includes Pohnpei STEPS 2002 only; 2005 Pohnpei STEPS data analysis and reporting not available; assessment not completed.

g Reports not available; assessment not completed.
